# Improved HER/OER Performance of NiS_2_/MoS_2_ Composite Modified by CeO_2_ and LDH

**DOI:** 10.3390/ma17194876

**Published:** 2024-10-04

**Authors:** Hao Li, Feng Chen, Xinyang Wu, Dandan Wang, Yongpeng Ren, Yaru Li

**Affiliations:** 1 Henan Key Laboratory of Green Building Materials Manufacturing and Intelligent Equipment, Luoyang Institute of Science and Technology, Luoyang 471023, China; lihao_2013@126.com; 2 School of Environmental and Biological Engineering, Henan University of Engineering, Zhengzhou 451191, China; chenfeng871588@163.com; 3 Henan Key Laboratory of High-Temperature Metal Structural and Functional Materials, National Joint Engineering Research Center for Abrasion Control and Molding of Metal Materials, Henan University of Science and Technology, Luoyang 471000, China; 13243386137@163.com (X.W.); 13343791260@163.com (D.W.); 4 Longmen Laboratory, Luoyang 471000, China

**Keywords:** transition-metal sulfides, layered double hydroxide, Cerium dioxide, OER, HER

## Abstract

In recent years, there has been significant interest in transition-metal sulfides (TMSs) due to their economic affordability and excellent catalytic activity. Nevertheless, it is difficult for TMSs to achieve satisfactory performance due to problems such as low conductivity, limited catalytic activity, and inadequate stability. Therefore, a catalyst with a heterostructure constituted of a nickel–iron-layered double hydroxide, nickel sulfide, molybdenum disulfide, and cerium dioxide was designed. At the current density of 10 mA cm^−2^ in an alkaline solution, the catalyst exhibits a HER overpotential of 116 mV. In addition, an overpotential of 235 mV@150 mA cm^−2^ was displayed for OER. The catalyst showed a good retention rate (94.7% for HER, 98.6% for OER) after 160 h stability tests. The excellent electrochemical performance is attributed to the following points: 1. The self-supporting three-dimensional hierarchical structure provides abundant sites, fast ion diffusion channels, and electron transfer paths, and ensures structural stability. 2. The strong interfacial electron interaction between Ni_3_S_2_/MoS_2_ heterojunction and NiFe-LDH improves the OER reaction kinetics. 3. The Ce^3+^ and oxygen vacancies in CeO_2_ promote the dissociation of H_2_O and promote the HER reaction kinetics. This approach paves the way for developing highly efficient electrocatalysts for various electrochemical applications.

## 1. Introduction

Nowadays, as industrial growth accelerates, the issues of energy scarcity and environmental pollution are escalating at an alarming rate. Due to its exceptional attributes such as high energy density, sustainability, cleanliness, and efficient heat release, hydrogen is widely regarded as a highly promising energy source [1,2]. Hydrogen production from water electrolysis is an effective method to produce hydrogen fuel, but the two half-reactions involved (hydrogen evolution reaction (HER), oxygen evolution reaction (OER)) face a huge reaction barrier, so Pt, Ir, and Ru-based precious metal catalysts are needed to accelerate the reaction kinetics [3]. The high cost and scarcity of noble metal-based materials limit the large-scale industrial application of electrolyzed water. Therefore, the design of non-precious metal catalysts with low cost, high activity, and strong stability has become the focus of research [4,5].

At present, the transition metal nickel sulfide/molybdenum disulfide composite (NiS_2_/MoS_2_) exhibits high activity for both HER and OER, and has become one of the most promising bifunctional electrocatalysts [6,7]. However, NiS_2_/MoS_2_ faces the problems of poor intrinsic activity, low conductivity, and insufficient stability of noble metals. Therefore, it is very important to develop efficient and stable transition metal matrix composite catalysts [8,9]. For example, Xu et al. synthesized a self-supporting heterostructure based on MoS_2_-CoFeLDH as an electrocatalyst for the complete decomposition of water in alkaline media. At 10 mA cm^−2^, the HER and OER of MoS_2_-CoFeLDH can reach 100 mV and 216 mV, respectively. The bifunctional catalyst requires a voltage of 1.55 V for total water decomposition and remains stable within 48 h. The strong electronegativity of LDH is beneficial to the adsorption of H, and the strong conductivity of MoS_2_ is beneficial to the desorption of H_2_ molecules [10,11,12]. Due to the strong adsorption capacity of LDHs for hydroxyl species, the addition of MoS_2_ promoted the migration of hydroxyl species, thus promoting the OER of the anode [13,14]. In their study, Li et al. synthesized a crystal-amorphous heterogeneous electrocatalyst, MoS_2_/NiVFe, by modifying MoS_2_ with a NiVFe-LDH using a two-step hydrothermal method. In alkaline and neutral media, at 10 mA cm^−2^, the HER and OER overpotentials are 78 and 141 mV, respectively, and the Tafel Slopes are 61.51 and 80.2 mV·dec^−1^, respectively. Crystal-amorphous heterostructures have been shown to provide a large electrochemically active surface area to enhance active sites and accelerate electron transfer. XPS and theoretical calculations reveal that the strong electron modulation effect of electron transfer from NiVFe-LDH to MoS_2_ leads to the adsorption of -OH at the high valence Ni site and the adsorption of -H at the electron-rich S site, which significantly reduces the potential barrier of water splitting [15,16,17,18].

In addition, doping rare earth elements is a viable approach to modify electron density and enhance the abundance of active sites. The unique 4f electronic structure of rare earth elements enables the coexistence of Ce^3+^ and Ce^4+^ oxidation states, facilitating surface oxygen ion exchange. The CeO_2_, known for its ample oxygen vacancies and strong affinity for oxygen-containing species, exhibits significant potential in electrocatalysis applications [19,20,21,22]. Tae-Hwan Oh developed a well-interconnected Ni_3_S_2_-MoS_2_-CeO_2_ nanostructure as a multifunctional electrocatalyst. Under alkaline conditions, the electrode exhibits significant oxygen evolution reaction (OER) performance, with a low overpotential (206.03 mV at 10 mA cm^−2^) and Tafel slope (40.19 mV·dec^−1^). The material also showed excellent stability, and the durability of the electrocatalyst was verified by chronopotentiometry. The inherent stability of CeO_2_ facilitates a robust and intimate electrochemical coupling with NiOOH. The combined presence of CeO_2_, MoS_2_, and NiOOH in the catalytic system leads to a synergistic interaction, resulting in improved catalytic performance [23,24,25]. Wu et al. developed and designed Ni_3_S_2_-CeO_2_ hybrid nanostructures as OER electrocatalysts by the electrodeposition method. Under 1.0 M KOH conditions, the Ni_3_S_2_-CeO_2_ electrode demonstrated a remarkable current density of 20 mA cm^−2^ with a low overpotential of 264 mV. This represents a decrease of 92 mV compared to the Ni_3_S_2_ alone. Additionally, the Tafel slope was measured at 146 mV·dec^−1^. The strong interfacial interaction between Ni_3_S_2_ and CeO_2_ greatly facilitates electron transfer within the Ni_3_S_2_ nanosheets, leading to enhanced water oxidation activity [26,27,28].

Based on the above considerations, LDH was introduced into NiS_2_/MoS_2_ transition metal sulfide to improve the OER performance of the composite material, and CeO_2_ modification was used to further promote the kinetics of hydrogen evolution and oxygen evolution reaction, so as to achieve low cost, high activity, and strong stability of the electrocatalyst to promote the performance of water electrolysis [29,30].

**Scheme 1:**
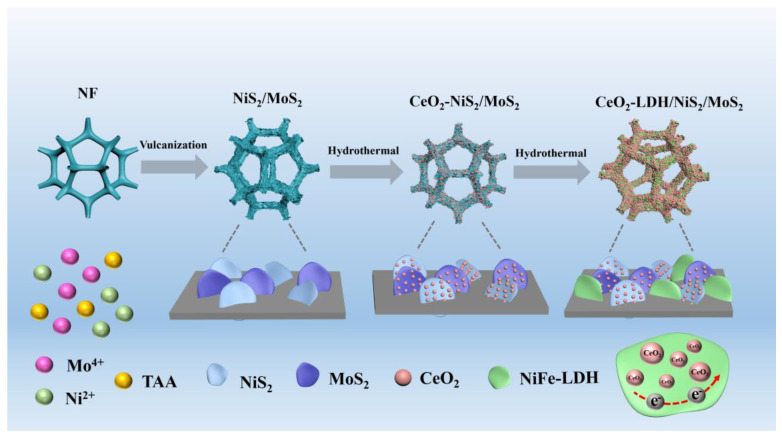
Preparation of CeO_2_-LDH/Ni_3_S_2_/MoS_2_.

Therefore, the CeO_2_-modified self-supporting hierarchical structure (CeO_2_-LDH/Ni_3_S_2_/MoS_2_) was designed and prepared on a three-dimensional nickel foam substrate by a two-step hydrothermal method for efficient OER and HER, as illustrated in Scheme 1. The CeO_2_-LDH/Ni_3_S_2_/MoS_2_ catalyst exhibits an overpotential of 116 mV at a current density of 10 mA cm^−2^ for HER. For the OER, it achieves an overpotential of 235 mV at 150 mA cm^−2^. The i-t curve of HER and OER did not decay significantly after 160 h of operation, showing good electrocatalytic stability. Ce doping at the optimal level in LDH structures can enhance catalytic activity by adjusting the electronic structure and local chemical binding environment, resulting in enhanced catalytic activity. In addition, the heterogeneous interface constructed by CeO_2_ modification promotes the structural evolution and charge transfer of the catalyst. Furthermore, LDHs’ electronic structure can be modulated and the change transfer during catalysis can be accelerated via redox interactions between Ce^3+^ and Ce^4+^. The Ni_3_S_2_/MoS_2_-NiFe-LDH composites facilitate strong interfacial charge transfer, leading to enhanced charge redistribution and improved reaction kinetics. Ce^3+^ and oxygen vacancies in CeO_2_ can promote the dissociation of H_2_O. CeO_2_ becomes the active site of HER and OER, and greatly improves the activity of hydrogen and oxygen evolution.

## 2. Experiment

### 2.1. Nickel Foam Pretreatment

A certain size of nickel foam (NF, 2 × 2 cm^2^) was cut, and the NF was ultrasonically cleaned in acetone and 3 M HCl for 15 min, in order to remove the grease and oxide layer on its surface. The nickel foam was washed with deionized water and anhydrous ethanol many times and then dried at 60 °C for 12 h.

### 2.2. Preparation of Ni_3_S_2_/MoS_2_

Ni_3_S_2_/MoS_2_ was prepared by one-step hydrothermal sulfidation. An amount of 0.0968 g of Na_2_MoO_4_·2H_2_O (Aladdin, Shanghai, China) and 0.0601 g of TAA (Aladdin, Shanghai, China) were put in deionized water and magnetically stirred for 30 min to obtain a clear and transparent mixed solution. The pretreated nickel foam was placed in the above solution and transferred to a stainless steel autoclave (Aladdin, Shanghai, China). The hydrothermal temperature was set at 200 °C in a blast drying oven for 22 h. After the reactor was cooled to room temperature, the nickel foam was washed several times with deionized water and vacuum-dried overnight. The product was named Ni_3_S_2_/MoS_2_.

### 2.3. Preparation of CeO_2_-Ni_3_S_2_/MoS_2_

The synthesis procedure of CeO_2_-Ni_3_S_2_/MoS_2_ was similar to that of Ni_3_S_2_/MoS_2_. On this basis, 0.0174 g of Ce (NO_3_)_3_·6H_2_O_2_ (Aladdin, Shanghai, China) was added. 

### 2.4. Synthesis of CeO_2_-LDH/Ni_3_S_2_/MoS_2_

CeO_2_-LDH/Ni_3_S_2_/MoS_2_ catalysts were prepared by a two-step hydrothermal method. Ni (NO_3_)_2_·6H_2_O (0.5452 g) (Aladdin, Shanghai, China), Fe(NO_3_)_3_·9H_2_O (0.2525 g) (Aladdin, Shanghai, China), NH_4_F (0.046 g) (Aladdin, Shanghai, China), and urea (0.375 g) (Aladdin, Shanghai, China) were dissolved in 25 mL deionized water and stirred by magnetic force for 30 min until the solution was clear and green. The CeO_2_-Ni_3_S_2_/MoS_2_ obtained in the third step was transferred together with the mixed solution to a 50 mL stainless steel autoclave with polytetrafluoroethylene lining, and reacted at 120 °C for 6 h in a blast drying oven. The product was named CeO_2_-LDH/Ni_3_S_2_/MoS_2_.

The preparation method of LDH/Ni_3_S_2_/MoS_2_ is the same as that of CeO_2_-LDH/Ni_3_S_2_/MoS_2_, except that Ce(NO_3_)_3_·6H_2_O was not added during the first hydrothermal step.

### 2.5. Material Characterization

The samples’ crystal structure was characterized using X-ray diffraction (XRD) with a Bruker X instrument (Bruker, Karlsruhe, Germany). The valence state and surface composition of the samples were simultaneously analyzed using X-ray photoelectron spectroscopy (XPS) with an ESCALAB 250 Xi instrument (Thermo Fisher, Waltham, MA, USA). The morphology of the samples was examined using field emission scanning electron microscopy (SEM) (Thermo Fisher, MA, USA) and transmission electron microscopy (TEM) with a JSM-IT800 and FEI Talos F200 s instrument (Thermo Fisher, MA, USA), respectively.

### 2.6. Electrode Preparation and Electrochemical Measurement

All electrochemical tests were performed on a Princeton electrochemical workstation with a three-electrode system (Aladdin, Shanghai, China) (reference electrode Ag/AgCl, graphite rod as counter electrode, working electrode). The catalyst supported or coated with nickel foam was used as the working electrode. The preparation of nickel foam-supported noble metal standard electrode is as follows: Pt/C or RuO_2_ powder samples were weighed and dispersed in 1 mL anhydrous ethanol, ultrasonicated for 10 min, and then 50 μL Nafion solution (5 wt%) was added and ultrasonicated for 25 min to obtain uniform black ink. These inks were evenly dropped on the NF and dried with a digital infrared baking lamp. All electrochemical tests were carried out in 1.0 M KOH (pH = 13.7) solution. All data in this paper are converted into data relative to the reversible hydrogen electrode according to the E_RHE_ = E_Ag/AgCl_0.197 + 0.0592 × pH–iR formula [31]. The OER and HER were characterized using linear sweep voltammetry (LSV) in a 1 M KOH solution at a scan rate of 5 mV s^−1^. The Tafel diagram is obtained by the equation ŋ = a + blog(j) [32]. The durability of the material was tested by chronoamperometry. In order to evaluate the electrochemical active surface area (ECSA) of the catalyst, the non-faradaic interval was selected, and the CV curves were drawn at the scanning rates of 60, 70, 80, 90, and 100 mV s^−1^, and the C_dl_ value was obtained by linear fitting. Electrochemical impedance spectroscopy (EIS) measurements were conducted over a frequency range of 100 kHz to 0.01 Hz, using an AC voltage amplitude of 10 mV. The stability of the catalyst was tested by chronoamperometry (i-t curve) at a constant potential.

## 3. Results and Discussion

### 3.1. Structure and Morphology Characterization

The synthesis process of CeO_2_-LDH/Ni_3_S_2_/MoS_2_ composites is shown in Scheme 1. In the first step, Ni_3_S_2_/MoS_2_ was prepared by one-step hydrothermal vulcanization using nickel foam as the nickel source, Na_2_MoO_4_·2H_2_O as the molybdenum source, and thioacetamide as the sulfur source. On this basis, CeO_2_-Ni_3_S_2_/MoS_2_ was prepared by the hydrothermal method by adding Ce(NO_3_)·6H_2_O. CeO_2_-LDH/Ni_3_S_2_/MoS_2_ was prepared by the hydrothermal method by adding Ni(NO_3_)_2_·6H_2_O, Fe(NO_3_)_3_·9H_2_O and urea.

**Figure 1:**
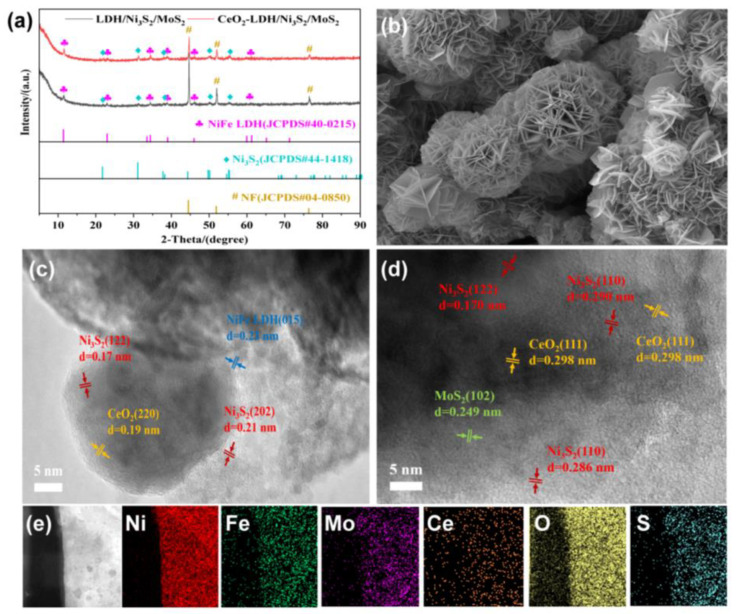
(**a**) XRD patterns of LDH/Ni_3_S_2_/MoS_2_ and CeO_2_-LDH/Ni_3_S_2_/MoS_2_, (**b**) SEM image of CeO_2_-LDH/Ni_3_S_2_/MoS_2_, (**c**,**d**) HRTEM images of CeO_2_-LDH/Ni_3_S_2_/MoS_2_. (**e**) Elemental mapping images of CeO_2_-LDH/Ni_3_S_2_/MoS_2_.

The structure of the composites was analyzed by XRD. As shown in the XRD pattern of Figure 1, the strong diffraction peaks at 44.4°, 51.8°, and 76.3° on the nickel foam (NF) substrate correspond to the (111), (200), and (220) crystal planes of NF, respectively. In the first step of the hydrothermal process, NF was inevitably sulfurized. The diffraction peaks at 21.8°, 31.1°, 37.7°, 44.4°, 49.8°, and 55.2° corresponded to the (101), (110), (003), (202), (113), and (122) crystal planes of Ni_3_S_2_ (JCPDS#No.44-1418), respectively. After the second step of hydrothermal treatment, new diffraction peaks appeared at 11.4°, 23.0°, 34.4°, 39.0°, 46.0°, 59.9°, and 61.3°, corresponding to the (003), (006), (012), (015), (018), (110), and (113) crystal planes of NiFe-LDH (JCPDS#No.40-0215). In addition, no diffraction peaks of CeO_2_ and MoS_2_ were observed in the XRD pattern, which may be due to the low content or the aggregation of MoS_2_ naturally accumulated during the hydrothermal process to a certain extent, resulting in poor crystallinity. Figure 1b and Figure S1 show that NiFe-LDH, LDH/Ni_3_S_2_/MoS_2,_ and CeO_2_-LDH/Ni_3_S_2_/MoS_2_ exhibit a flower-like shape composed of nanosheet structures. The nanosheets grown on the nickel foam are relatively uniform, smooth, and densely covered. Figure S1b,e are two different sheet morphologies of LDH/Ni_3_S_2_/MoS_2_, showing a more dispersed growth mode than NiFe-LDH (Figure S1a,d), forming a larger flower ball. In this transition, the vertically growing and spatially interconnected network is well maintained, suggesting that this structure helps to expose more catalytically active sites, provide open channels for electrolyte accessibility, and facilitate the rapid release of bubbles. In addition, Figure S1c,f show that CeO_2_-modified LDH/Ni_3_S_2_/MoS_2_ is uniformly grown on nickel foam, and the cross-linking of nanosheets in different growth directions leads to a more open porous structure. This not only makes the catalyst have a large surface area, which is conducive to the penetration of the electrolyte, but also makes the catalyst have strong permeability. The morphological characteristics of CeO_2_-LDH/Ni_3_S_2_/MoS_2_ materials were characterized by high-resolution transmission electron microscopy. The test results shown in Figure 1c,d are high-resolution transmission images. In the HRTEM images, the lattice fringes of 0.170 nm, 0.290 nm, 0.286 nm, and 0.21 nm are from the (122), (100), (110), and (202) crystal planes of Ni_3_S_2_, respectively. The lattice fringe of 0.249 nm corresponds to the (102) crystal plane of MoS_2_, and the lattice fringes of 0.298 nm and 0.19 nm are from the (111) and (220) crystal planes of CeO_2_. In addition, there are 0.23 nm lattice fringes corresponding to the (015) crystal plane of NiFe-LDH, which confirms the successful preparation of CeO_2_-LDH/Ni_3_S_2_/MoS_2_. Figure 1c confirms the lamellar structure. The surface scanning results of the elements show that Ni, Fe, Mo, Ce, O, and S are uniformly distributed in the CeO_2_-LDH/Ni_3_S_2_/MoS_2_ catalyst (Figure 1e). The results of XRD, SEM, and TEM prove that the CeO_2_-LDH/Ni_3_S_2_/MoS_2_ hierarchical structure material is successfully synthesized.

**Figure 2:**
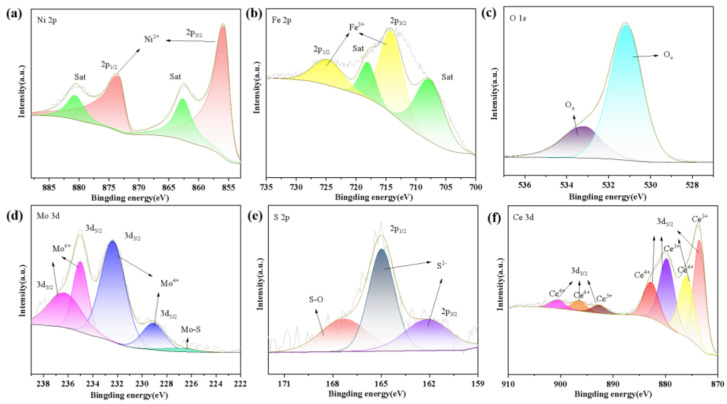
High-resolution XPS spectrogram of CeO_2_-LDH/Ni_3_S_2_/MoS_2_: (**a**) Ni 2p, (**b**) Fe 2p, (**c**) O 1s, (**d**) Mo 3d, (**e**) S 2p, (**f**) Ce 3d.

The surface composition and valence state of the samples are analyzed based on XPS. The XPS total spectrum of Figure S2 demonstrates the presence of Ni, Fe, O, Mo, S, and Ce elements in CeO_2_-LDH/Ni_3_S_2_/MoS_2_. As shown in Figure 2a, in the XPS spectrum of Ni 2p, the Ni 2p energy level is split into 2p_1/2_ and 2p_3/2_, corresponding to the binding energy of 873.42 and 855.81 eV, respectively, showing Ni^2+^. The satellite peaks are located at 880.65 and 862.62 eV, respectively. At this time, no metal Ni peak was observed, which is due to the uniform growth of NiFe LDH nanosheets on Ni_3_S_2_/MoS_2_, so it is difficult to detect Ni^0^ from nickel foam [33]. For the Fe 2p (Figure 2b) spectrum, two peaks at 724.7 and 714.09 eV could be attributed to Fe 2p_1/2_ and Fe 2p_3/2_, respectively, indicating the existence of the Fe^3+^ oxidation state. The other two satellite peaks correspond to the binding energies of 718 and 707.67 eV [34]. These results prove the formation of NiFe-LDH. The O 1s spectrum (Figure 2c) shows two peaks at 531.17 and 533.13 eV, which are related to M-OH and H-O-H in layered dioxygen, respectively, proving the presence of oxygen vacancies (O_v_) and adsorbed oxygen (O_a_) [35]. The oxygen vacancies in the material are conducive to the adsorption and dissociation of H_2_O and thus improve the electrocatalytic performance. Figure 2d displays the spectrum of Mo 3d, which has two characteristic peaks at 236.29 and 235 eV, corresponding to 3d_3/2_ and 3d_5/2_ of Mo^6+^, respectively. There are two characteristic peaks at 232.5 and 229.07 eV, corresponding to 3d_3/2_ and 3d_5/2_ of Mo^4+^, respectively. Another characteristic peak is 227.02 eV, which can be attributed to the formation of the Mo-S bond [36]. In Figure 2e, the XPS spectra of the S 2p region display binding energies of 164.98 and 162.13 eV, corresponding to S 2p_1/2_ and S 2p_3/2_, respectively, which are attributed to the metal sulfides corresponding to S^2−^ and the strong S-O peak (167.32 eV) due to the oxidation of sulfides [37]. In the Ce 3d spectrum (Figure 2f), the peaks at 900.61 eV, 896.45 eV, and 892.63 eV are the characteristic peaks of Ce^4+^ 3d_3/2_, Ce^4+^ 3d_3/2,_ and Ce^3+^ 3d_3/2_, respectively. The peaks at 882.74 eV, 879.74 eV, 875.97 eV, and 873.51 eV are the characteristic peaks of Ce^4+^ 3d_5/2_, Ce^3+^ 3d_5/2_, Ce^4+^ 3d_5/2,_ and Ce^3+^ 3d_5/2_, respectively [38]. The CeO_2_ exhibits a flexible transition between the Ce^3+^ and Ce^4+^ oxidation states, which contributes to its excellent electronic and ionic conductivity, reversible oxygen ion exchange at the surface, and high oxygen storage capacity. These inherent properties make CeO_2_ a valuable co-catalyst, facilitating efficient charge transport and enhancing energy efficiency. Consequently, CeO_2_ plays a crucial role in enhancing the activity of water oxidation catalysts [39,40,41].

### 3.2. Electrochemical HER Performance

**Figure 3:**
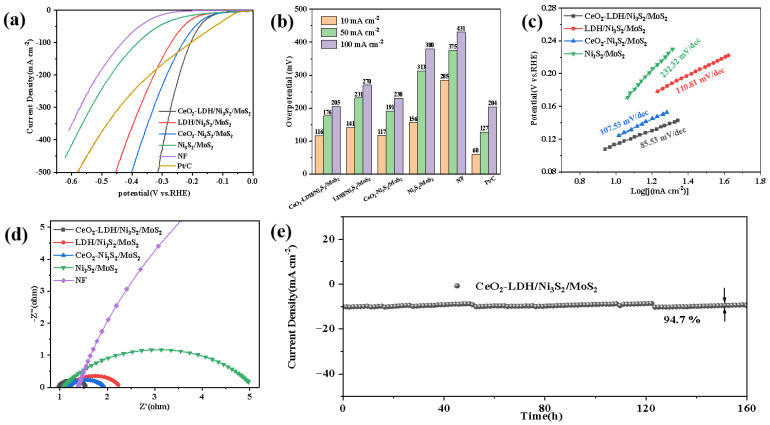
HER electrocatalytic performance in 1.0 M KOH: (**a**) LSV curve, (**b**) overpotential, (**c**) Tafel slope, (**d**) EIS spectrum, (**e**) durability of CeO_2_-LDH/Ni_3_S_2_/MoS_2_ determined by chronoamperometry at a current density of 10 mA cm^−2^.

Based on the three-electrode system, the hydrogen evolution performance of the catalyst was studied in an alkaline medium (1.0 M KOH). The HER performance of different samples is shown in Figure 3a,b, and the single nickel foam has almost no HER performance. At a current density of 10 mA cm^−2^, the overpotential of CeO_2_-LDH/Ni_3_S_2_/MoS_2_ is 116 mV, lower than that of Ni_3_S_2_/MoS_2_ (156 mV), CeO_2_-Ni_3_S_2_/MoS_2_ (117 mV), LDH/Ni_3_S_2_/MoS_2_ (141 mV), and commercial precious metal Pt/C (60 mV). With the current density increased to 50 and 100 mA cm^−2^, the overpotentials of Ni_3_S_2_/MoS_2_, CeO_2_-Ni_3_S_2_/MoS_2_, LDH/Ni_3_S_2_/MoS_2_, CeO_2_-LDH/Ni_3_S_2_/MoS_2,_ and commercial precious metal Pt/C were 313 and 380 mV, 191 and 230 mV, 231 and 270 mV, 176 and 205 mV, and 127 and 204 mV, respectively. It is confirmed that the combination of LDH and two-component transition metal sulfides can slightly improve the hydrogen evolution performance. CeO_2_ nanoparticles can effectively enhance the hydrogen evolution activity of transition metal sulfides. The experimental results show that the noble metal Pt/C has the best performance at low current density. With the increase in current density, the catalytic performance of CeO_2_-LDH/Ni_3_S_2_/MoS_2_ exhibits the best activity. It is proved that the CeO_2_-LDH/Ni_3_S_2_/MoS_2_ electrode is suitable for high current density.

In order to further understand the kinetics of the reaction, the Tafel slope curves of different samples were obtained according to the LSV curve. As shown in Figure 3c, the Tafel slope value of CeO_2_-LDH/Ni_3_S_2_/MoS_2_ is 85.53 mV dec^−1^, which is lower than that of LDH/Ni_3_S_2_/MoS_2_ (110.81 mV dec^−1^), CeO_2_-Ni_3_S_2_/MoS_2_ (107.53 mVdec^−1^), and Ni_3_S_2_/MoS_2_ (232.3 mVdec^−1^). This indicates that CeO_2_-LDH/Ni_3_S_2_/MoS_2_ has the optimal HER kinetics. In addition, in order to further understand the charge transfer resistance of the catalyst material, an EIS test was performed on the prepared material. The charge transfer resistance R_ct_ value of CeO_2_-LDH/Ni_3_S_2_/MoS_2_ is 0.57 Ω, less than that of LDH/Ni_3_S_2_/MoS_2_ (1.06 Ω), CeO_2_-Ni_3_S_2_/MoS_2_ (0.79 Ω), Ni_3_S_2_/MoS_2_ (4.00 Ω), and pure NF(33.28 Ω), indicating that the introduction of Ce accelerates the electron transfer at the interface between the catalyst and the electrolyte [42,43,44].

Stability is also one of the important factors to evaluate the catalyst. Figure 3e shows the HER chronoamperometry curve for evaluating the electrochemical stability of CeO_2_-LDH/Ni_3_S_2_/MoS_2_. During the electrolysis process, the electrode can remain stable for 160 h. The curve showed no obvious attenuation. The current density was maintained at 10 mA cm^−2^ with a retention rate of 94.7%, which proved that the CeO_2_-LDH/Ni_3_S_2_/MoS_2_ electrode maintained excellent catalytic activity and good stability [45,46,47].

### 3.3. Electrochemical OER Performance

**Figure 4:**
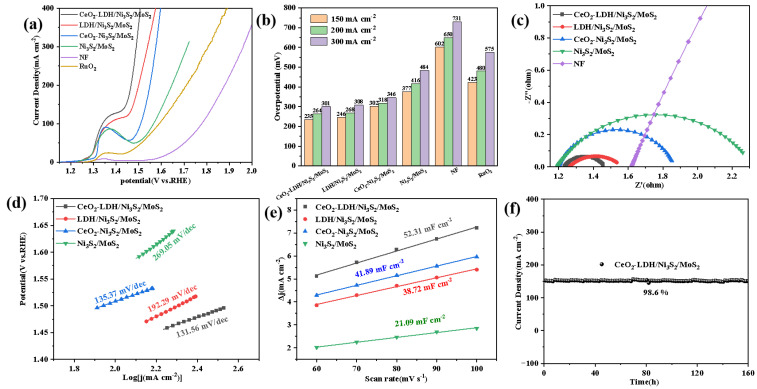
OER electrocatalytic performance in 1.0 M KOH: (**a**) LSV curve, (**b**) overpotential, (**c**) Tafel slope, (**d**) EIS spectrum, (**e**) the C_dl_, (**f**) durability of CeO_2_-LDH/Ni_3_S_2_/MoS_2_ determined by chronoamperometry at a current density of 150 mA cm^−2^.

Based on the three-electrode system, the oxygen evolution performance of the catalyst was studied in an alkaline medium (1.0 M KOH). The results are shown in Figure 4a,b. There is almost no OER activity on the nickel foam substrate, but due to the three-dimensional porous structure and metal activity of nickel foam, there is an obvious oxidation peak at about 1.35 V, which proves the excellent properties of nickel foam as a skeleton material [48,49]. At a current density of 150 mA cm^−2^, the overpotential of CeO_2_-LDH/Ni_3_S_2_/MoS_2_ is 235 mV, lower than that of LDH/Ni_3_S_2_/MoS_2_ (246 mV), CeO_2_-Ni_3_S_2_/MoS_2_ (302 mV), and Ni_3_S_2_/MoS_2_ (377 mV), and even better than that of commercial noble metal RuO_2_ (423 mV). The overpotentials of Ni_3_S_2_/MoS_2_, CeO_2_-Ni_3_S_2_/MoS_2_, LDH/Ni_3_S_2_/MoS_2_, and CeO_2_-LDH/Ni_3_S_2_/MoS_2_ are 416, 484, 318, 346, 268, and 308 mV, respectively, as the current densities increased to 200 and 300 mA cm^−2^.

The intrinsic catalytic kinetics of the material was further evaluated by the Tafel slope curve. As shown in Figure 4c, the Tafel slopes of CeO_2_-LDH/Ni_3_S_2_/MoS_2_, LDH/Ni_3_S_2_/MoS_2_, CeO_2_-Ni_3_S_2_/MoS_2,_ and Ni_3_S_2_/MoS_2_ are 131.56,192.29,135.37, and 269.05 mA cm^−2^, respectively. The CeO_2_-LDH/Ni_3_S_2_/MoS_2_ shows the lowest Tafel slope, which proves optimal reaction kinetics and further confirms excellent OER catalytic activity [50,51]. In addition, the charge transfer resistance of the interface between the electrocatalyst and the electrolyte was studied by testing the Nyquist curve in EIS. The semicircle diameter reflects the rate of electron transfer. The smaller the arc radius of the Nyquist curve, the smaller the charge transfer resistance R_ct_ value. As shown in Figure 4d, the R_ct_ values of LDH/Ni_3_S_2_/MoS_2_ (0.30 Ω), CeO_2_-Ni_3_S_2_/MoS_2_ (0.67 Ω), and Ni_3_S_2_/MoS_2_ (1.13 Ω) indicate that the CeO_2_-LDH/Ni_3_S_2_/MoS_2_ composite structure grown on NF has the smallest charge transfer resistance and the highest kinetic reaction rate, which is also consistent with the kinetic results of Tafel slope [52,53]. 

ECSA was used to evaluate the number of active sites of the catalyst. Since the ECSA is linearly proportional to the electrochemical double-layer capacitance (C_dl_), the C_dl_ is commonly used to indirectly compare the size of the electrochemically active surface area. Figure S3 shows the variable speed CV curves of different catalysts. As can be seen in Figure 4e, the C_dl_ of the CeO_2_-LDH/Ni_3_S_2_/MoS_2_ electrode is 52.31 mF cm^−2^, which is higher than that of LDH/Ni_3_S_2_/MoS_2_ (38.72 mF cm^−2^), CeO_2_-Ni_3_S_2_/MoS_2_ (41.89 mF cm^−2^), and Ni_3_S_2_/MoS_2_ (21.09 mF cm^−2^), indicating that the CeO_2_-LDH/Ni_3_S_2_/MoS_2_ electrode combines LDH and CeO_2_ active components to expose more catalytic active sites, and the introduction of LDH increases the contact with water molecules. Thus, the OER catalytic activity is significantly improved [54]. 

Figure 4f shows the stability curve of the CeO_2_-LDH/Ni_3_S_2_/MoS_2_ composite electrode running for 160 h at 150 mA cm^−2^. It can be seen that the current of the catalyst is stable and smooth in the alkaline electrolyte, and the percentage of current density change is 98.6%, indicating good electrocatalytic stability. This is attributed to the corrosion resistance and good conductivity of the nickel foam substrate [55]. Additionally, the comparable reported catalysts of OER and HER are shown in Table S2 and Table S3, respectively.

### 3.4. Overall Water Splitting

**Figure 5:**
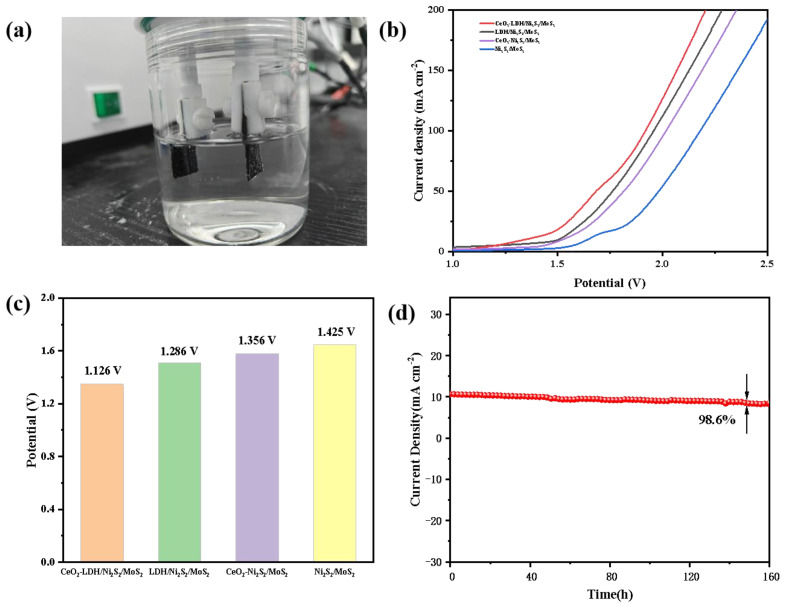
The two-electrode electrolyzers for overall water splitting of CeO_2_-LDH/Ni_3_S_2_/MoS_2_||CeO_2_-LDH/Ni_3_S_2_/MoS_2_, LDH/Ni_3_S_2_/MoS_2_||LDH/Ni_3_S_2_/MoS_2_, CeO_2_-Ni_3_S_2_/MoS_2_||CeO_2_-Ni_3_S_2_/MoS_2,_ and Ni_3_S_2_/MoS_2_||Ni_3_S_2_/MoS_2_ in 1.0 M KOH. (**a**) The digital photograph of the O_2_ and H_2_ evolution from CeO_2_-LDH/Ni_3_S_2_/MoS_2_. (**b**) The polarization curves, (**c**) overpotentials at 10 mA cm^−2^, and (**d**) chronopotentiometry test at 10 mA cm^−2^ for CeO_2_-LDH/Ni_3_S_2_/MoS_2_||CeO_2_-LDH/Ni_3_S_2_/MoS_2_.

In order to evaluate the electrocatalytic performance of overall water splitting, we constructed a two-electrode electrolyzer using CeO_2_-LDH/Ni_3_S_2_/MoS_2_ as both the anode and cathode in a 1.0 M KOH solution (Figure 5a). The results from Figure 5b,c demonstrate that the CeO_2_-LDH/Ni_3_S_2_/MoS_2_||CeO_2_-LDH/Ni_3_S_2_/MoS_2_ electrolyzer achieved a cell voltage of 1.126V at 10 mA cm^−2^, which is significantly lower than that of LDH/Ni_3_S_2_/MoS_2_ (1.286 V), CeO_2_-Ni_3_S_2_/MoS_2_ (1.356 V), and Ni_3_S_2_/MoS_2_ (1.574 V). Moreover, Figure 5d reveals that the CeO_2_-LDH/Ni_3_S_2_/MoS_2_||CeO_2_-LDH/Ni_3_S_2_/MoS_2_ electrode maintained 98.6% of its initial activity after operating for 160 h at 10 mA cm^−2^. These results validate the excellent durability of the CeO_2_-LDH/Ni_3_S_2_/MoS_2_ composite for overall water splitting in an alkaline electrolyte. Additionally, the comparisons of OER and HER activities between Ni_3_S_2_/MoS_2_-based electrocatalysts recently reported are displayed in Table S1 and Table S2, respectively. The CeO_2_-LDH/Ni_3_S_2_/MoS_2_ electrolyzer displayed a comparable cell voltage to that of the Ni_3_S_2_/MoS_2_-based electrocatalysts, as shown in Table S3.

## 4. Conclusions

In summary, we have successfully prepared a CeO_2_ and LDH co-modified hierarchical structure (CeO_2_-LDH/Ni_3_S_2_/MoS_2_) as a novel non-precious metal electrocatalyst by a two-step hydrothermal method. The CeO_2_-LDH/Ni_3_S_2_/MoS_2_ catalyst was tested for electrochemical hydrogen evolution and oxygen evolution under alkaline conditions. It was proved that the HER overpotential of CeO_2_-LDH/Ni_3_S_2_/MoS_2_ was 116 mV at a current density of 10 mA cm^−2^, and the OER overpotential was only 235 mV at 150 mA cm^−2^. In addition, the CeO_2_-LDH/Ni_3_S_2_/MoS_2_||CeO_2_-LDH/Ni_3_S_2_/MoS_2_ electrolyzer achieved a cell voltage of 1.126V at 10 mA cm^−2^. Moreover, the CeO_2_-LDH/Ni_3_S_2_/MoS_2_||CeO_2_-LDH/Ni_3_S_2_/MoS_2_ electrode maintained 98.6% of its initial activity after operating for 160 h at a current density of 10 mA cm^−2^.

The catalyst exhibits excellent HER/OER performance due to the LDH, which increases the oxygen evolution active site, promotes the contact between the electrode and the alkaline electrolyte, and accelerates the OER reaction kinetics. In addition, CeO_2_ modifies the local electronic structure, which is conducive to the interaction between electrons and accelerates the transfer of charges, thereby improving the electrocatalytic performance of OER/HER. CeO_2_ also promotes the change of morphology. The hierarchical structure composed of nickel foam, nanosheets, and nanoparticles increases the active sites, which is beneficial for the improvement of electrocatalytic performance. According to the activity of the catalyst, it can be used for water decomposition in the future to decompose water into hydrogen and oxygen, thereby achieving an environmentally friendly energy conversion process. Compared with traditional fossil fuel combustion, this technology can reduce a large number of greenhouse gas emissions and air pollutants. This work provides a new idea for the design of highly active and stable electrocatalytic materials.

## Data Availability

The data generated during and/or analyzed in this article are available from the corresponding author upon reasonable request.

## Supplementary Materials

The following supporting information can be downloaded at: https://www.mdpi.com/article/10.3390/ma17194876/s1, Figure S1: SEM of (a,d) LDH, (b,e) LDH/Ni_3_S_2_/MoS_2_, (c,f) CeO_2_-LDH/Ni_3_S_2_/MoS_2_, Figure S2: XPS survey spectra of CeO_2_-LDH/Ni_3_S_2_/MoS_2_, Figure S3: Cyclic voltammograms (CVs) from 60 to 100 mV/s for (a) Ni_3_S_2_/MoS_2_, (b) CeO_2_-Ni_3_S_2_/MoS_2_, (c) LDH-Ni_3_S_2_/MoS_2_, (d) CeO_2_-LDH/Ni_3_S_2_/MoS_2_ at the different scan rates, Table S1: Comparison of OER catalytic activities between Ni_3_S_2_/MoS_2_ and other recently reported OER electrocatalysts in both 1 M KOH; Table S2: Comparison of HER catalytic activities between Ni_3_S_2_/MoS_2_ and other recently reported HER electrocatalysts in both 1 M KOH, Table S3: Comparison of water splitting performance between Ni_3_S_2_/MoS_2_ and other recently reported electrocatalysts in both 1 M KOH.
