# Supplementary material for: Improved HER/OER Performance of NiS2/MoS2 Composite Modified by CeO2 and LDH
Source: Materials (Basel). 2024 Oct 4;17(19):4876. doi: 10.3390/ma17194876 (PMC11478180; doi:10.3390/ma17194876)
Supplement: Supplementary file 1 [file materials-17-04876-s001.zip › materials-3195541-supplementary.pdf]

materials

Supplementary Materials

Article

Improved HER/OER Performance of NiS_2/MoS_2 Composite Modified by CeO_2 and LDH

Hao Li [1,†], Feng Chen [2,†], Xinyang Wu [3], Dandan Wang [3], Yongpeng Ren [3,4,*] and Yaru Li [3,4,*]

1 Henan Key Laboratory of Green Building Materials Manufacturing and Intelligent, Luoyang Institute of Science and Technology, Luoyang 471023, China; lihao_2013@126.com
2 School of Environmental and Biological Engineering, Henan University of Engineering, Zhengzhou 451191, China; chenfeng871588@163.com
3 Henan Key Laboratory of High-temperature Metal Structural and Functional Materials, National Joint Engineering Research Center for Abrasion Control and Molding of Metal Materials, Henan University of Science and Technology, Luoyang 471000, China; 13243386137@163.com (X.W.); 13343791260@163.com (D.W.)
4 Longmen Laboratory, Luoyang 471000, China
* Correspondence: ren_yp123@163.com (Y.R.); jiayouli138@163.com (Y.L.)
† These authors contributed equally to this work.

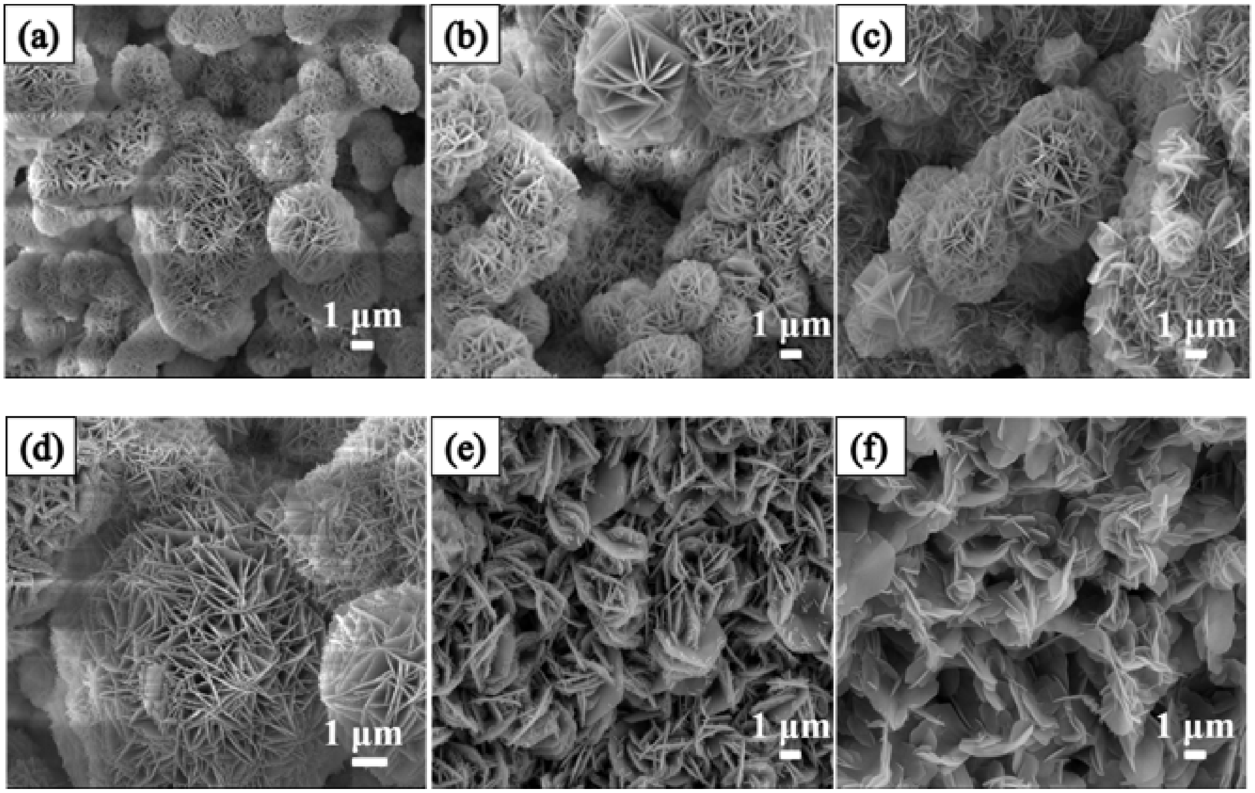

Figure S1. SEM of (a, d) LDH, (b,e) $LDH/Ni_3S_2/MoS_2$, (c,f) CeO_2-$LDH/Ni_3S_2/MoS_2$.

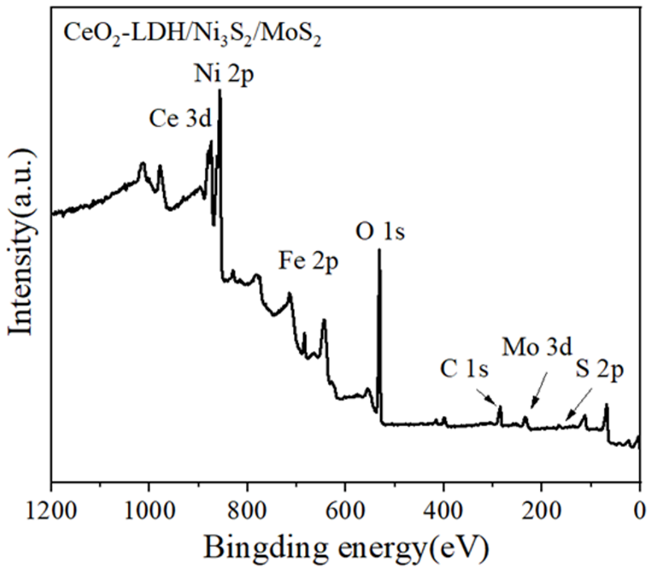

Figure S2. XPS survey spectra of CeO_2-LDH/Ni_3S_2/MoS_2.

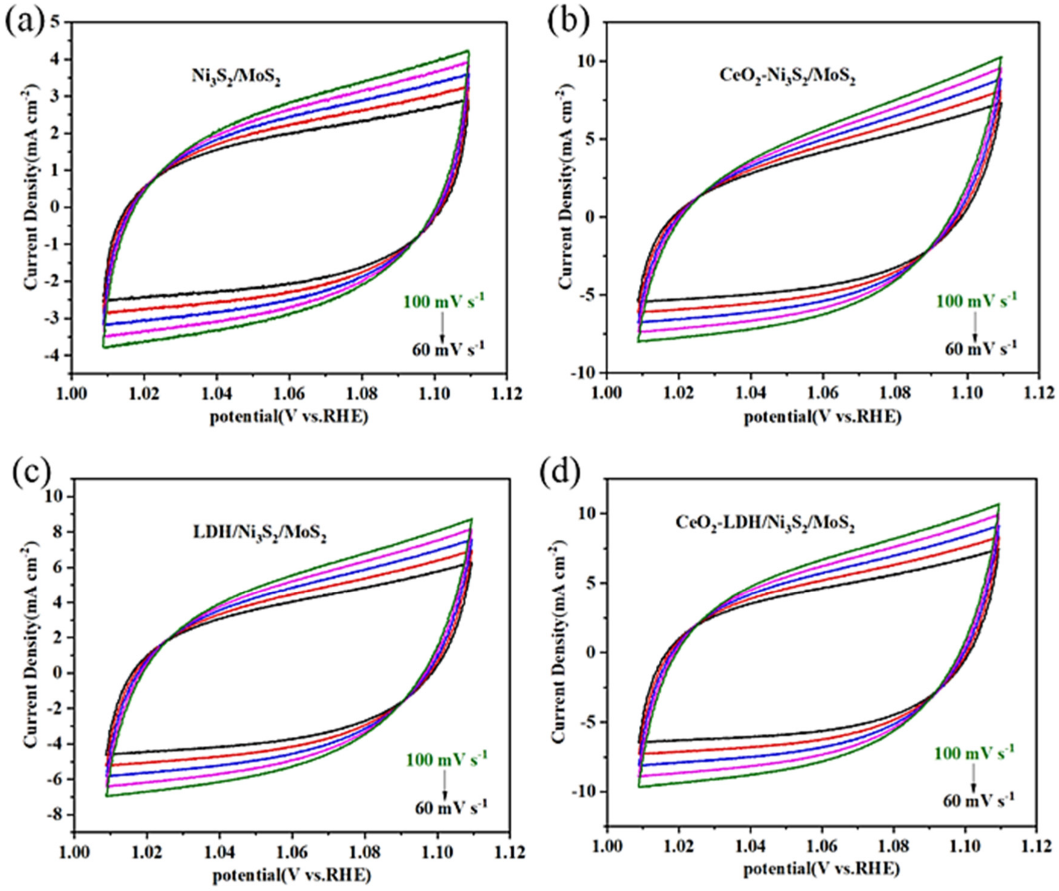

Figure S3. Cyclic voltammograms (CVs) from 60 to 100 mV/s for (a) Ni_3S_2/MoS_2, (b) CeO_2-Ni_3S_2/MoS_2, (c) LDH-Ni_3S_2/MoS_2, (d) CeO_2-LDH/Ni_3S_2/MoS_2 at the different scan rates.

Table S1. Comparison of OER catalytic activities between Ni_3S_2/MoS_2 and other recently reported OER electrocatalysts in both 1 M KOH.

Electrocatalysts	Current density (mA/cm²)	Voltage (mV)	Reference
CeO_2-LDH/Ni_3S_2//MoS_2	150	235	This work
Ce-CoFe-LDH/NF	10	225	[1]
Fe,Ce-Ni_3S_2/NiS/NF	100	234	[2]
Ce-$Co(OH)_2$/CoP/NF	10	253	[3]
P,Ce-$FeNi_3N$/NF	200	340	[4]
CeFeCoP/NF	50	298	[5]
Co–MoS_2/Ni_3S_2-GO/NF	10	123	[6]
MoS_2/NiCoS/NF	10	220	[7]
$CoNi_2S_4$/Ni_3S_2@NF	10	243	[8]
NF@G-5@Ni_3S_2	10	249	[9]

Table S2. Comparison of HER catalytic activities between Ni_3S_2/MoS_2 and other recently reported HER electrocatalysts in both 1 M KOH.

Electrocatalysts	Current density (mA/cm²)	Voltage (mV)	Reference
CeO_2-LDH/Ni_3S_2//MoS_2	10	116	This work
Ce-$Co(OH)_2$/CoP/NF	10	56	[3]
CeFeCoP/NF	10	97	[5]
Co–MoS_2/Ni_3S_2-GO/NF	10	161	[6]
MoS_2/NiCoS/NF	10	90	[7]
$CoNi_2S_4$/Ni_3S_2@NF	10	171	[8]
NF@G-5@Ni_3S_2	10	119	[9]
NiO/MoS_2/NF	10	121	[10]
MoS_2/NiCoP/NF	10	148	[11]

Table S3. Comparison of water splitting performance between Ni_3S_2/MoS_2 and other recently reported electrocatalysts in both 1 M KOH.

Electrocatalysts	Current density (mA/cm²)	Voltage (V)	Reference
CeO_2-LDH/Ni_3S_2//MoS_2	10	1.348	This work
Ce-$Co(OH)_2$/CoP/NF	10	1.53	[3]
CeFeCoP/NF	10	1.55	[5]

Co–MoS_2/Ni_3S_2-GO/NF	10	1.47	[6]
MoS_2/NiCoS/NF	10	1.49	[7]
$CoNi_2S_4/Ni_3S_2$@NF	10	1.65	[8]
NF@G-5@Ni_3S_2	10	1.62	[9]

Reference

1. Lu, W.; Yi, L.; Wei, X. C. 3D nanostructured Ce-doped CoFe-LDH/NF self-supported catalyst for high-performance OER. *Dalton transactions* **2023**, *52 (34),* 12038-12048.
2. Wang, S.; Li, H.; Li, S.; Ni, Y. Fe,Ce Co-Doped Ni3S2/NiS Polymorphism Nanosheets with Improved Electrocatalytic Activity and Stability for Water Oxidation. *ChemSusChem* **2024**, e202400896, https://doi.org/10.1002/cssc.202400896.
3. Lyu, C.; Cheng, J.; Wu, K.; Wu, J.; Hao, J.; Chen, Y.; Wang, H.; Yang, Y.; Wang, N.; Lau, W.-M.et al. Interface and cation dual-engineering promoting Ce-Co$(OH)_2$/CoP/NF as bifunctional electrocatalyst toward overall water splitting coupling with oxidation of organic compounds. Journal of Alloys and Compounds 2023, 934, 167942.
4. Li, S.; Du, Y.; Wang, M.; Liu, J.; Li, B.; Gu, Y.; Wang, L. Optimizing the reaction pathway of nitride electrode by co-doping strategy for boosting alkaline hydrogen evolution reaction kinetics. *Sci. China Mater.* **2023**, *66*, 4639–4649, https://doi.org/10.1007/s40843-023-2632-y.
5. Shen, L.; Tang, S.; Yu, L.; Huang, Q.; Zhou, T.; Yang, S.; Yu, H.; Xiong, H.; Xu, M.; Zhong, X.; et al. Efficient ternary CeFeCoP bifunctional electrocatalyst for overall water splitting. *J. Solid State Chem.* **2022**, *314*, https://doi.org/10.1016/j.jssc.2022.123434.
6. Yao, Y.; He, J.; Zhu, X.; Mu, L.; Li, J.; Li, K.; Qu, M. Wettability and heterojunction synergistic interface optimization guided Co doped MoS_2/Ni_3S_2-GO/NF catalytic electrode to boost overall water splitting. International Journal of Hydrogen Energy 2024, 51, 207-221.
7. Xu, X.; Zhong, W.; Zhang, L.; Liu, G.; Xu, W.; Zhang, Y.; Du, Y. NiCo-LDHs derived NiCo2S4 nanostructure coated by MoS2 nanosheets as high-efficiency bifunctional electrocatalysts for overall water splitting. *Surf. Coatings Technol.* **2020**, *397*, 126065, https://doi.org/10.1016/j.surfcoat.2020.126065.
8. Dai, W.; Ren, K.; Zhu, Y. A.; Pan, Y.; Lu, T. Flower-like $CoNi_2S_4/Ni_3S_2$ nanosheet clusters on nickel foam as bifunctional electrocatalyst for overall water splitting. Journal of Alloys and Compounds 2020, 844, 156252.
9. Jin, C.; Zhou, N.; Wang, Y.; Li, X.; Chen, M.; Dong, Y.; Yu, Z.; Liang, Y.; Qu, D.; Dong, Y.; et al. 3D porous and self-supporting Ni foam@graphene@Ni3S2 as a bifunctional electrocatalyst for overall water splitting in alkaline solution. *J. Electroanal. Chem.* **2019**, *858*, 113795, https://doi.org/10.1016/j.jelechem.2019.113795.
10. Xia, K.; Cong, M.; Xu, F.; Ding, X.; Zhang, X. Targeted Assembly of Ultrathin NiO/MoS_2 Electrodes for Electrocatalytic Hydrogen Evolution in Alkaline Electrolyte. Nanomaterials 2020, 10 (8), 1547.
11. Minmin, W.; Hao, X.; Yanfeng, T. T. Heterogeneous electrocatalysts of MoS_2/NiCoP for highly stable hydrogen evolution. New Journal of Chemistry 2024, 48 (3), 1200-1205.
